# Supplementary material for: New algorithms for accurate and efficient de novo genome assembly from long DNA sequencing reads
Source: Life Sci Alliance. 2023 Feb 22;6(5):e202201719. doi: 10.26508/lsa.202201719 (PMC9946810; doi:10.26508/lsa.202201719)
Supplement: Supplementary file 4 [file LSA-2022-01719_TableS4.docx]

Supplementary Table T4. PacBio assembly configuration parameters

| Genome | Tool | Version | Parameters |
| --- | --- | --- | --- |
| Rice | NGSEP | 4.3.1 | -k 25 -w 40 |
| Maize | NGSEP | 4.3.1 | -k 25 -w 40 |
| CHM13 | NGSEP | 4.3.1 | -k 25 -w 40 |
| HG002 | NGSEP | 4.3.1 | -k 25 -w 40 -ploidy 2 |
| Rice | Canu | 2.1.1 | -pacbio-hifi -genomeSize=360m |
| Maize | Canu | 2.1.1 | -pacbio-hifi -genomeSize=2.1g |
| CHM13 | Canu | 2.1.1 | -pacbio-hifi -genomeSize=3.1g |
| HG002 | Canu | 2.1.1 | -pacbio-hifi -genomeSize=3.1g |
| Rice | Flye | 2.8.3 | -pacbio-hifi -g 360m |
| Maize | Flye | 2.8.3 | -pacbio-hifi -g 2.1g |
| CHM13 | Flye | 2.8.3 | -pacbio-hifi -g 3.1g |
| HG002 | Flye | 2.8.3 | -pacbio-hifi -g 3.1g |
| Rice | HiFiASM | 0.15 | -l0 |
| Maize | HiFiASM | 0.15 | -l0 |
| CHM13 | HiFiASM | 0.15 | -l0 |
| HG002 | HiFiASM | 0.15 | default |
