# Supplementary material for: New algorithms for accurate and efficient de novo genome assembly from long DNA sequencing reads
Source: Life Sci Alliance. 2023 Feb 22;6(5):e202201719. doi: 10.26508/lsa.202201719 (PMC9946810; doi:10.26508/lsa.202201719)
Supplement: Supplementary file 5 [file LSA-2022-01719_TableS5.docx]

Supplementary Table T5. Nanopore assembly configuration parameters

| Genome | Tool | Version | Parameters |
| --- | --- | --- | --- |
| *E. coli* | NGSEP | 4.3.1 | -k 15 -w 30 |
| *S. cerevisiae* | NGSEP | 4.3.1 | -k 15 -w 30 |
| *D. melanogaster* | NGSEP | 4.3.1 | -k 15 -w 30 |
| CHM13 | NGSEP | 4.3.1 | -k 25 -w 40 |
| *E. coli* | Canu | 2.1 | -nanopore genomeSize=4.8m -correctedErrorRate=0.039 |
| *S. cerevisiae* | Canu | 2.1 | -nanopore genomeSize=12m |
| *D. melanogaster* | Canu | 2.1 | -nanopore genomeSize=139m |
| *E. coli* | Flye | 2.8.3 | --nano-raw --genome-size 4.8m |
| *S. cerevisiae* | Flye | 2.8.3 | --nano-raw --genome-size 12m |
| *D. melanogaster* | Flye | 2.8.3 | --nano-raw --genome-size 139m |
| *E. coli* | NECAT | 0.0.1 | GENOME_SIZE=4800000 |
| *S. cerevisiae* | NECAT | 0.0.1 | GENOME_SIZE=12000000 CNS_OUTPUT_COVERAGE=40  FSA_OL_FILTER_OPTIONS=-min_coverage 3 |
| *D. melanogaster* | NECAT | 0.0.1 | GENOME_SIZE=139000000 |
| CHM13 | NECAT | 0.0.1 | GENOME_SIZE=3000000000 |
